# *In vitro* BMP2 stimulation of osteoblast citrate production in concert with mineralized bone nodule formation

**DOI:** 10.7243/2050-1218-4-2

**Published:** 2015-10-17

**Authors:** Leslie C. Costello, Meenakshi A. Chellaiah, Jing Zou, Mark A. Reynolds, Renty B. Franklin

**Affiliations:** 1 Department of Oncology and Diagnostic Sciences, Dental School, University of Maryland, Baltimore, USA; 2 Department of Periodontics, Dental School, University of Maryland, Baltimore, USA

**Keywords:** BMP2, citrate production, ZIP1 transporter, mineralized bone formation, osteoblasts, apatite/collagen complex, osteogenesis, citration and mineralization, mesenchyme stem cells

## Abstract

**Background:**

That citrate is a major indispensible component of bone in humans and in all osteovertebrates has been known for about seventy-five years. Yet, its role and importance in the structure and function of bone and bone formation have remained unknown. However, recent studies have identified that citrate is a major and essential component of the apatite/collagen structure of bone; and that the biomechanical properties of bone (e.g., stability, strength, resistance to fracture) depend on the appropriate incorporation of citrate in the structure of bone. The osteoblasts have recently been identified as citrate-producing cells that provide the citrate that is incorporated in the apatite/collagen structure during osteogenesis. Little is known regarding the factors and mechanisms involved in the regulation of citrate that is incorporated along with mineralization during the process of bone formation. Because of the importance of BMP2 in the initiation of osteogenesis and the development of the osteoblasts, it is essential to determine its possible implication in the development of the citrate-producing capability of the osteoblasts (i.e., “citration”) during the formation of mineralized bone nodules.

**Methods:**

The goal of this study was to determine if BMP2 promotes the development of citrate-producing osteoblasts for increased citrate incorporation in the formation of mineralized bone nodules. The study employed MC3T3 mesenchyme stem cell osteogenic differentiation in the presence and absence of BMP2.

**Results:**

The results showed that BMP2 treatment increased the osteogenic development of mineralized bone nodules. In addition, BMP2 increased osteoblast citrate production and incorporation in the mineralized bone nodule. This was accompanied by increased ZIP1 transporter, which is an essential genetic/metabolic event for citrate-producing cells.

**Conclusions:**

The results demonstrate, for the first time, that BMP2 facilitates the osteoblast “citration” process in concert with mineralization during bone formation; and provide confirmation of the important role of osteoblasts as specialized citrate-producing cells in the process of bone formation. However, it is essential to determine if these *in vitro* effects will occur *in vivo* in BMP2-implant induction of bone formation. “Citration” is essential for osteoinductive bone to represent the chemical, structural, and biomechanical properties of “normal” bone.

## Introduction

High levels of citrate constitute a major component of bone (and teeth) in humans and in all osteovertebrates. It comprises ~1.6 % of the bone content; ~5% of the organic component of bone; and ~80% of the total body citrate resides in bone. The fact that all osteovertebrates exhibit this high bone citrate composition (cartilage does not contain the high citrate levels) is evidence that citrate is an indispensible essential component of bone. Although this has been known since 1941, the implications of citrate in bone have received little attention or recognitions over the past~35 years. Consequently, progress and advances in the identification and elucidation of citrate relationships in normal bone and in bone disorders remain largely unknown. However, recent studies [1–4] have identified that citrate is an important component of the apatite/collagen structure of bone; and it is essential to achieve optimal manifestation of the important biomechanical properties of bone (such as stability, strength, resistance to fracture).

This important role of citrate now brings attention to the necessity for increased research into citrate implications in normal bone formation and in bone disorders. One of the unresolved fundamental issues has been the identification of the source of citrate for incorporation into bone. We recently identified [5,6] that the osteoblasts are specialized citrate-producing cells, which provide the citrate incorporation (i.e., “citration”), along with mineralization, during bone formation. The osteoblast metabolic and functional capability occurs during osteogenic differentiation of the mesenchyme stem cells. This is a new understanding of the role of osteoblasts and also the events of osteogenesis and bone formation.

Now it becomes essential to identify the factors and events that regulate osteoblast citrate production and incorporation into bone. BMP2 is important for initiating and optimizing early osteogenic events leading to bone formation, including mineralized bone nodule formation during mesenchyme stem cells differentiation [7–9]. Therefore, we initiated studies to determine if BMP2 also stimulated osteoblast citrate production and “citration” of mineralized bone nodules in osteogenic differentiation of MC3T3 cells.

## Methods

### Cell culture

Mouse mesenchyme stem cells (MC3T3) were obtain from American Type Culture Collection (ATCC, Manassas, VA). The mesenchyme cells were grown and maintained in mesenchymal cell growth medium which consisted α-MEM supplemented with L-glutamine and containing 30 mg/ml gentamicin, 15 ug/ml amphotericin and 10% fetal bovine serum. Cells were cultured at 37°C in the presence of 5% CO_2_ until ~75% confluent. The mesenchyme cells were induced to differentiate to the osteoblast linage by culturing the cells in growth medium containing ascorbic acid (50 mg/ml) and B-glycerophosphate (10 mM). For BMP2 treatment, cells were maintained in osteogenic medium containing 100 ng/ml BMP2 with media changes every two days. Control cells were maintained in the osteogenic medium without BMP2.

### Alizarin red staining

Mineralization was assessed by staining with Alizarin Red for calcium deposits. Briefly, the medium was aspirated from the wells and the osteoblast cells and bone nodules fixed by incubation in iced cold 70% ethanol for 1 hour. The ethanol was removed and the wells rinsed twice with water. The water was then removed and enough Alizarin Red Solution (40 mM) added to cover the wells. After 30 min the Alizarin Red solution was removed and the wells washed four times with water. Mineralization was documented using an inverted microscope with a Qicam Fas1394 digital camera.

### Citrate and calcium assay

For the determination of citrate and calcium production, the medium was aspirated from the wells, and replaced by a volume of PBS. The PBS was removed by aspiration, followed by the addition of lysing buffer. The content of the well was scraped and mixed in the lysing buffer. The lysate was sonicated and deproteinized by addition of 7% trichroloacetic acid and centrifugation at 300g for 5 min. The citrate concentration of the lysate extract was measured by the fluoroenzymatic method previously described [10]. Calcium concentration in the lysate extract was assayed using the Calcium Colorimetric Assay kit from BioVision, Inc.

### Western blot analysis

Osteoblast cells were lysed using RIPA buffer (Upstate Biotech). The protein concentration of the lysates was determined using the BioRad protein assay based on the method of Bradford [11]. Proteins were separated by SDS-PAGE and transferred to nitrocellulose membrane. The membranes were blocked by incubating in PBS containing 5% non-fat milk and 0.1% tween 20 for 2 hours at room temperature. ZIP1 was detected by incubating the membranes overnight with ZIP1 chicken polyclonal antibody as described previously [12]. ZIP1 bound antibody was detected by incubating the membranes with hydrogen peroxide labeled goat anti-chicken secondary antibody and enhanced chemiluminescence detection reagents. The membranes were stripped and re-probed using anti β-actin antibody.

### Experimental and statistical analyses

The experiment was repeated so as to establish reproducibility of the results obtained. The results presented below are representative of the repeated experiments. In each experiment, the control and experimental groups were prepared in triplicate. T-test determination of the mean±sem values was employed to determine statistical significance.

## Results

**Figure 1:**
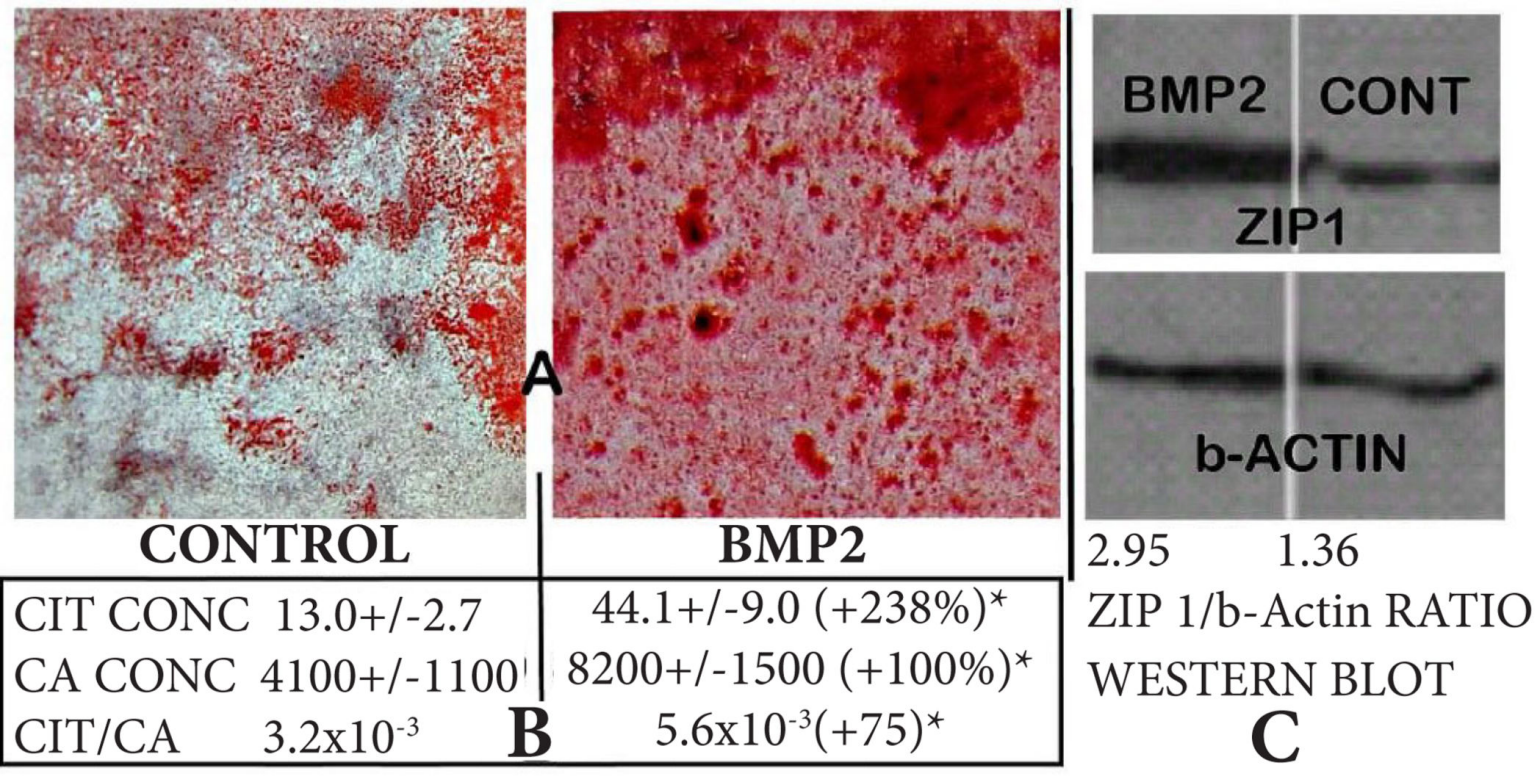
Effects of BMP2 on MC3T3 cell osteogenic differentiation leading to mineralized bone nodule formation. **A**. Alizarin red stain of wells showing effects of BMP2 on formation of mineralized bone nodules. **B**. Calcium and citrate levels of the cellular and bone nodule composition of the wells. **C**. Western blot showing BMP2 effect on ZIP1 transporter abundance. MC3T3 mesenchyme cells cultured in osteogenic medium+5uM Zn+50uM ASP (25 days). Control=no BMP2; BMP2=100ng/ml.

We first determined the effects of BMP2 on mineralized bone nodule formation resulting from osteogenic differentiation of MC3T3 cells. Alizarin Red staining reveals the apparent BMP2-induced increase in mineralized nodule formation (Figure 1A). This is corroborated by the quantitative determination of calcium which shows a 100% increase in calcium incorporated into the BMP2 treated cultures (Figure 1B). These results are consistent with the well-established role of BMP2 in enhancing the osteogenic differentiation of mesenchyme stem cells resulting in osteoblast mineralization during bone formation. The determination of citrate (Figure 1B) shows that BMP2 markedly increased (+238%) the level of citrate production and incorporation compared to the control. Most importantly, the citrate/calcium ratio was significantly increased (+75%) by BMP2 treatment. This reveals that in addition to its affect on increasing the differentiation of osteoblasts resulting in mineralized bone nodules, BMP2 additionally increases osteoblast citrate production and incorporation during mineralized bone formation (i.e., the “citration” of bone). Prior to differentiation, the undifferentiated MSCs exhibit very little citrate production (~5 nmols/mg protein) and calcium incorporation (~360 nmols/mg protein) compared to the respective concentrations following differentiation (see Figure 1B for values of the control and BMP2 treated differentiated cultures).

Since the upregulation of ZIP1 expression is required for optimal osteogenic differentiation of human mesenchyme stem cells leading to mineralizing and citrate producing osteoblasts [6,13], we determined if this relationship was evident in the BMP2 effects. Figure 1C shows the marked increase in ZIP1 transporter abundance in response to BMP2 treatment. Along with this, we obtained an increase in zinc levels (umols/mg protein) from 0.20±0.06 (control) to 0.29±0.03 for BMP2 treatment; an increase of 45%. However, the difference was not significant due to the small sample size. Nevertheless, the upregulation during differentiation of the MC3T3 cells is consistent with the observations obtained with human mesenchyme stem cell differentiation.

## Discussion

**Figure 2:**
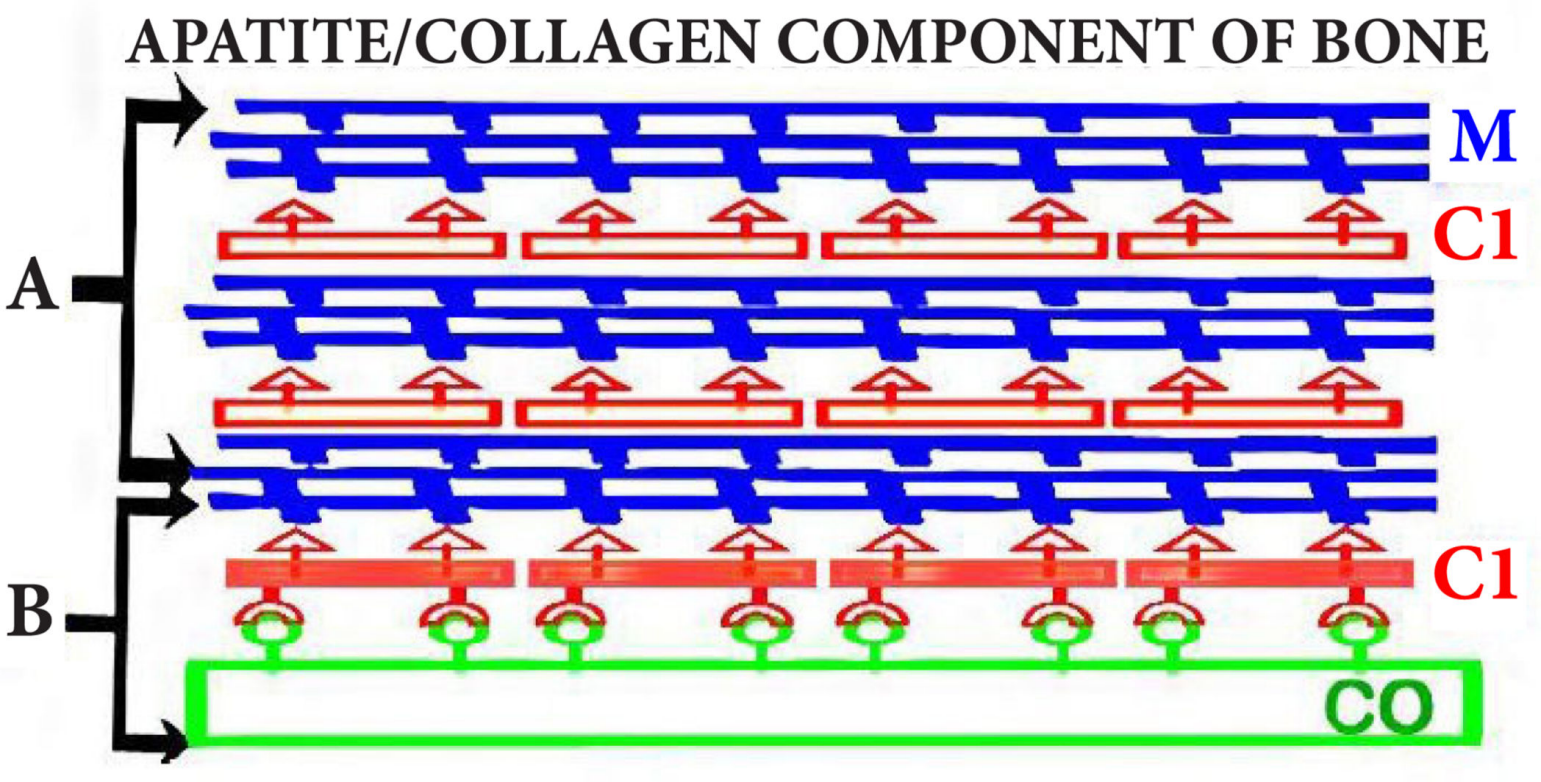
The status of citrate in the apatite/collagen component of bone. **A**. The apatite component. **B**. The apatite/collagen complex. M: Mineral; CI: Citrate; CO: Collagen.

The effects of BMP2 in this study must be discussed in relation to the new and more appropriate understanding of the implications of citrate in the composition and structure of normal bone formation. Until recently, descriptions of the apatite/collagen structure of bone focused on the mineralization of collagen; with no inclusion of citrate as a major component of the apatite/collagen complex. Recent studies have identified that citrate is incorporated in the structure of the apatite/collagen complex; and that it is essential for bone to exhibit its important biomechanical properties [1–4]. Figure 2 provides a representation of the apatite/collagen structure of bone; in which citrate is incorporated in the apatite nanoncrystal (mineral) component, and in the apatite/collagen complex [4]. Heretofore, this essential incorporation of citrate in the structure of bone had not been appropriately represented in the contemporary view of the structure of bone.

**Figure 3:**
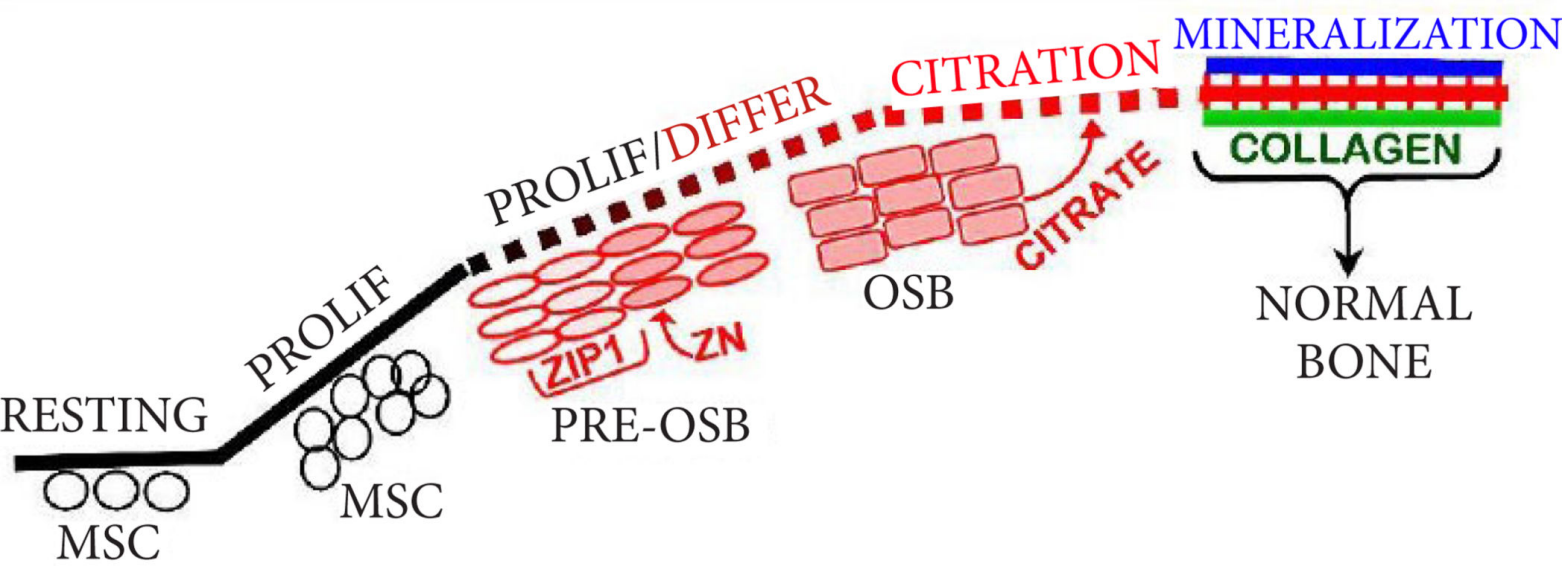
Normal bone formation showing osteogenic differentiation and the role of OSB citrate production and “citration” in concert with the formation of the apatite/collagen complex. Also shows the ZIP1 transporter upregulation and zinc uptake requirement for differentiation to OSB citrate-producing cells.

This leads to the new understanding that the process of bone formation must include the incorporation of citrate (which we refer to as “citration”) along with mineralization in the apatite nanocrystal/collagen complex. This also implies that the source of the citrate during bone formation must be identified. Our recent studies with human mesenchyme cells and primary osteoblasts [5,6] identified the osteoblasts as specialized “citrate-producing” cells. Under appropriate conditions, osteogenic mesenchyme stem cell differentiation results in osteoblasts with citrate-producing as well as mineralizing capabilities. In this study, the osteogenic differentiation of the MC3T3 cells (in the absence of added BMP2) resulting in osteoblast citrate production along with formation of mineralized bone nodules corroborates and extends our earlier studies. In addition, the concurrent BMP2-induced increase in citrate production and increased mineralized bone nodules provides additional evidence that osteoblast citrate production and “citration” are coordinated events in bone formation. Therefore, Figure 3 provides the new and more appropriate representation of osteogenesis during bone formation. In the absence of citration, the bone will not exhibit its important structural and biomechanical properties; and is not representative of “normal” bone.

That BMP2 is an important factor in inducing the differentiation of stem cells or precursor cells into osteoblastic lineages is well established [7–9,14,15]; although the downstream factors associated with the development of the functional osteoblasts and the early formation of mineralized bone nodules during the bone formation process remain largely unknown. In this study, the BMP2-induced increase in osteoblast mineralized bone nodule formation (Figure 1) is representative of the reported BMP2 induction of osteoblast differentiation and mineralization. However, the importance of BMP2 is additionally revealed by its specific induction of osteoblast citrate production (i.e., increased citration), in addition to increasing mesenchyme stem cell differentiation and development of the osteoblasts.

**Figure 4:**
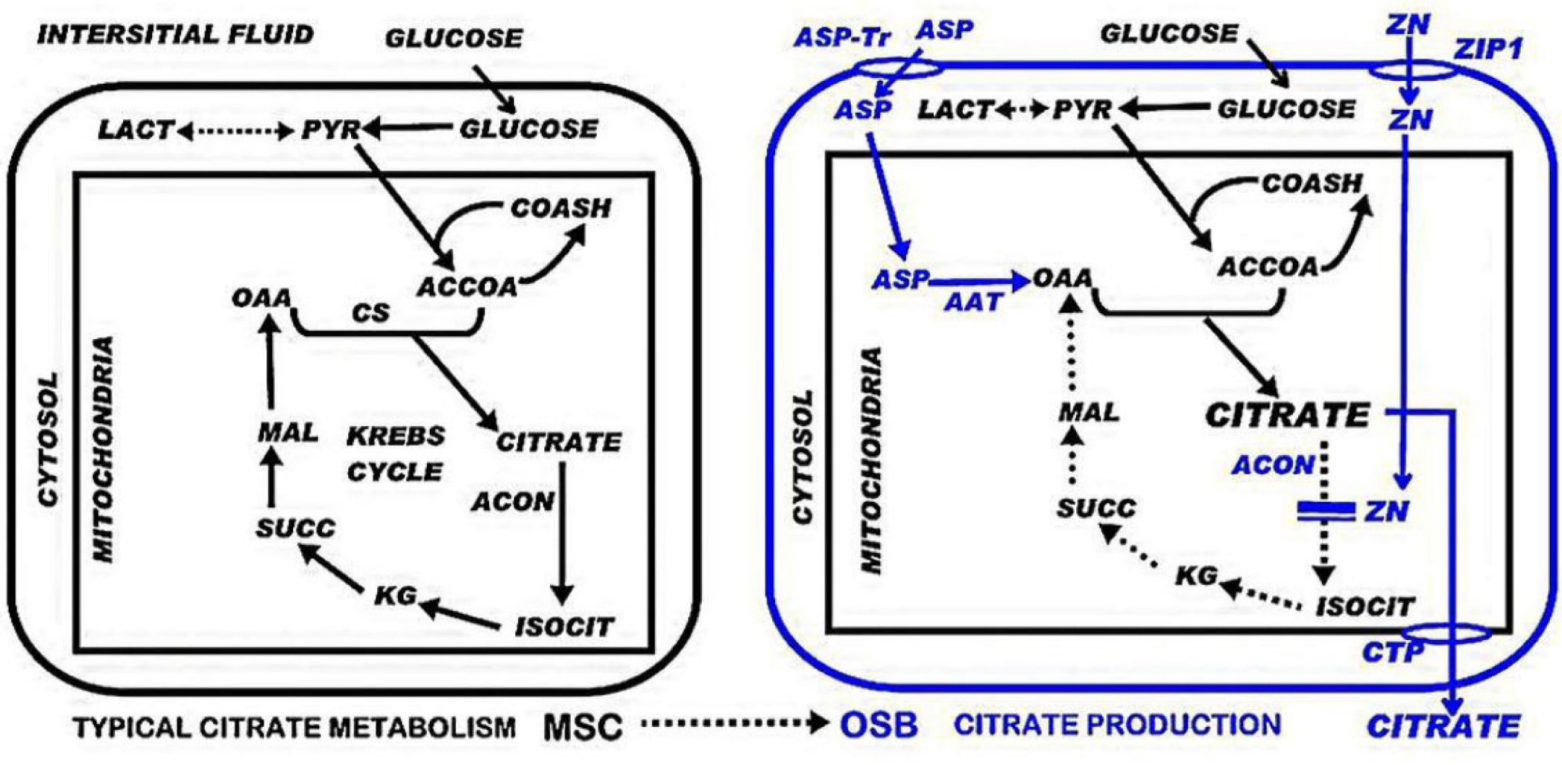
Important genetic/metabolic alterations (in blue) for the differentiation of mesenchyme stem cells to citrate-producing osteoblasts. ASP-Tr: Aspartate transporter; ZIP1: Zinc uptake transporter; CTP: Citrate transport protein; AAT: Aspartate aminotransferase; ACON: Aconitase.

The mechanisms by which BMP2 facilitates osteoblast citrate production requires an understanding of the unique cellular metabolic relationships involved in “net citrate production”; which is a specialized functional metabolic activity that does not typically exist in mammalian cells (Figure 4). The cellular metabolic pathway of net citrate production has been identified by our studies with prostate epithelial cells, which are specialized citrate-producing cells [16]. This functional metabolic relationship now applies to osteoblasts. It requires genetic/metabolic changes in the typical mammalian cell citraterelated intermediary metabolism; such as represented by the osteogenic differentiation transformation of mesenchymal cells to citrate-producing osteoblasts [5,6,17] (Figure 4). The most important initiating metabolic event is the increased cellular/mitochondrial concentration of zinc, which results in the specific inhibition of m-aconitase [18]. This is essential so that the citrate that is synthesized in the mitochondria is not converted to isocitrate and oxidized via the Krebs cycle; but instead, the citrate is accumulated for its transport out of the mitochondria and secreted into the extracellular environment. The increase in zinc uptake is achieved by the upregulation of ZIP1 zinc uptake transporter (Slc39A1).

Therefore, it is especially significant that the BMP2-induced increased citrate production is accompanied by increased ZIP1 transporter abundance (Figure 1C), which increases zinc accumulation; and is the major genetic/metabolic event for the osteoblasts to achieve net citrate production. However, other genetic/metabolic alterations as represented in Figure 4 are also required for osteoblast citrate production; and some of these might be induced by BMP2. Tang et al., [13] reported that optimal osteogenic differentiation of human MSC to mineralizing osteoblasts requires an accompanying increase in ZIP1 expression; and that the increased ZIP1 expression leads to altered expression of other genes that are implicated in the osteogenic process. This included the upregulation of mAAT, which is another genetic/metabolic alteration required for net citrate production (Figure 4). Thus, these BMP2-induced effects are consistent with the other events required for the osteogenic differentiation of human mesenchyme cells [7–9,14,15]. It is now important to elucidate the mechanism, factors, and signaling pathway(s) associated with BMP2 regulation of osteoblast citrate production.

**Figure 5:**
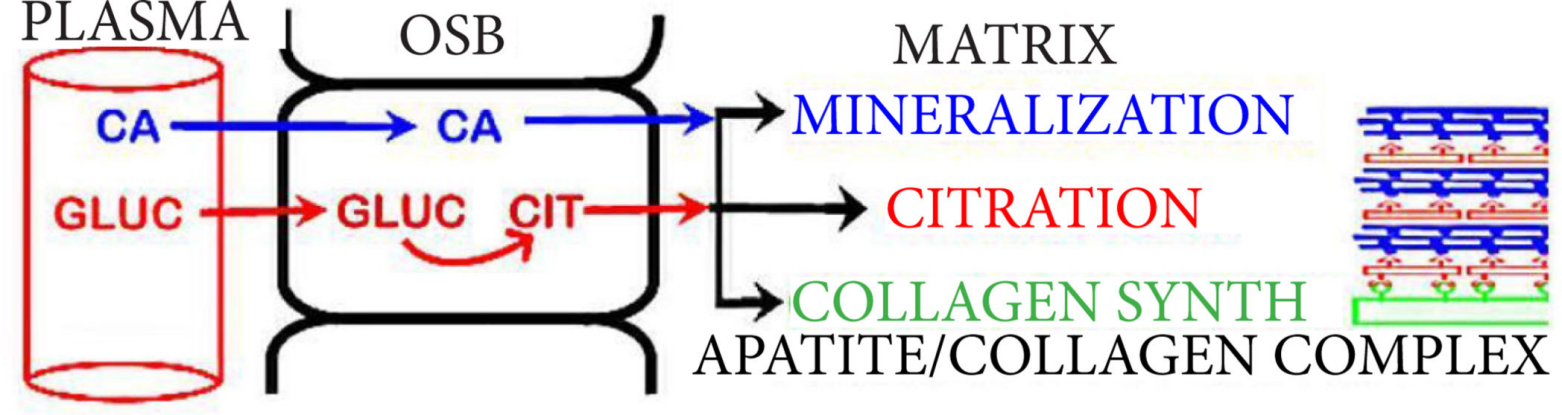
Osteoblast de novo citrate production; and calcium transport from plasma for incorporation into the a patite/collagen structure in the process of bone formation.

It must also be recognized that the incorporation of calcium and the incorporation of citrate in the formation of the bone nodules result from different sources and processes (Figure 5). The calcium is derived by the osteoblast uptake and transport from the calcium component of the extracellular medium. In contrast, the extracellular medium is devoid of citrate. Therefore, osteoblast de novo synthesis of citrate provides the citrate incorporated into the mineralized bone nodules. So, it becomes apparent that BMP2 stimulates osteoblast de novo citrate production. Furthermore, the results demonstrate that an exogenous extracellular source of citrate, unlike calcium, is not required for citrate incorporation during bone formation. The long-held prevailing view of plasma citrate homeostasis and bone turnover has represented that citrate, along with calcium, is transported into bone during bone formation. Based on our studies [5,6], that view is no longer tenable; and this impacts the understanding of hormonal/humoral actions implicated in the homeostatic regulation of citrate, calcium, bone and plasma.

The important implications of citrate should be considered in relation to the application of regenerative medicine and implant technology for osteoinductive treatment of bone disorders and repair of bone defects. The optimal bone formation from such treatments should be the generation of new bone that exhibits the chemical, structural, and biomechanical properties of the “normal” bone. Presently, no information or reported studies exist in which the presence or status of citrate has been determined or considered in implant-induced bone formation. The results of this study now implicate BMP2 in facilitating osteoblast citration in addition to facilitating osteoblast differentiation. Since BMP2 is osteoinductive *in vivo*, it is important to determine if BMP2 implants result in “citrated” mineralized bone formation, so as to exhibit the composition and biomechanical properties as exists in normal bone formation.

Most importantly, the results of this study, along with recent reports [1–6], provide compelling evidence of the essential and indispensible requirements of citrate incorporation to achieve normal bone formation. As such, the absence of recognition and consideration of the role of citrate in bone over the past forty years should be remedied by renewed interest and research into the implications of citrate in bone. This is essential to achieve the appropriate and accurate understanding of the process and factors involved in normal bone formation; the implications in the development of bone disorders; and the translation for the treatment of bone disorders and defects.
